# Adolescent girls' experiences of menstruation and schooling in monastic schools in Magway Region, Myanmar: A mixed-methods exploration

**DOI:** 10.3389/frph.2022.893266

**Published:** 2022-08-01

**Authors:** Zay Yar Swe, Nwe Oo Mon, Kyu Kyu Than, Peter S. Azzopardi, Elissa C. Kennedy, Jessica Davis, Lia J. Burns, Julie Hennegan

**Affiliations:** ^1^Myanmar Country Program, International Development Discipline, Burnet Institute, Yangon, Myanmar; ^2^University of Maryland School of Public Health, College Park, MD, United States; ^3^Global Adolescent Health Group, Maternal Child and Adolescent Health Program, Burnet Institute, Melbourne, VIC, Australia; ^4^Adolescent Health and Wellbeing Program, Aboriginal Health Equity Theme, South Australian Health and Medical Research Institute, Adelaide, SA, Australia; ^5^Department of Paediatrics, School of Medicine Dentistry and Health Sciences, University of Melbourne, Melbourne, VIC, Australia; ^6^School of Public Health and Preventive Medicine, Monash University, Melbourne, VIC, Australia; ^7^Independent Consultant, Melbourne, VIC, Australia; ^8^ChildFund Vietnam, Hanoi, Vietnam; ^9^Melbourne School of Population and Global Health, University of Melbourne, Melbourne, VIC, Australia

**Keywords:** menstrual health, menstrual hygiene, adolescent girls, survey, adolescent health, education

## Abstract

**Background:**

Despite increasing recognition that menstruation matters for adolescent girls' health and education, few studies have investigated menstrual health challenges and impacts in Myanmar. In this study we aimed to (1) understand the menstrual experiences of girls attending monastic schools in Magway Region, Myanmar and (2) explore the associations between their reported unmet menstrual health needs and school absenteeism.

**Methods:**

We undertook a mixed-methods exploration across 16 Monastic schools in rural and semi-rural areas. In-depth interviews with 10 adolescent girls, 10 Focus-Group Discussions (FGDs) with girls, 10 FGDs with boys, 5 FGDs with mothers, along with 24 key-informant interviews were analyzed using a framework approach to explore girls' menstrual experiences and challenges in school settings. A cross-sectional survey of 421 post menarche girls (mean-age-14 years) was used to describe the prevalence of menstrual health challenges and test associations with self-reported school absenteeism.

**Results:**

Girls described a range of menstrual health challenges including access to information and social support, behavioral restrictions, stigma surrounding menstruation, difficulties managing menstrual bleeding and pain. Girls also described fear and distress associated with menstruation and impacts on school attendance and participation. Of girls surveyed, 12.8% had missed school due to their last period. In multivariable analysis, grade level (aOR = 0.76 95%CI 0.60–0.97), menstrual pain (aOR = 2.10 95%CI 1.10–4.00), and heavy bleeding (aOR = 3.33 95%CI 1.51–7.34) were associated with absenteeism. Knowledge about menstrual biology was not related to absenteeism, but a more negative attitude toward menstruation may have predicted greater absences (aOR 1.34 95%CI 0.99–1.80). Confidence to talk to friends or teachers about menstruation was not associated with absenteeism, nor was using a disposable-pad or feeling confident to manage menses at school. However, feeling confident to ask a teacher for a pad was associated with greater absenteeism and may have indicated that girls more regularly needing to request products had lower attendance (aOR = 1.93 95%CI 1.06–3.54).

**Conclusions:**

Adolescent girls in Magway face substantial challenges during menstruation, adversely impacting on their education and wellbeing. Providing age-appropriate education and addressing shame and taboos are important components of a comprehensive menstrual health response. In addition, our study highlights the need to ensure access to menstrual resources and WASH facilities, along with access to adequate menstrual pain relief.

## Introduction

Menstruation is a pubertal milestone. Yet, this natural monthly occurrence is challenging for many girls and women worldwide (1). An estimated 500 million women and girls lack what they need to manage their menstruation (2). Across low- and middle-income countries in particular, these unmet menstrual needs present barriers to gender equality, with consequences for women's and girls' lives including physical and psychological ill-health, education including school absenteeism, employment, and social engagement (1).

Qualitative studies across countries have highlighted numerous challenges that girls face during menstruation. Many girls lack access to knowledge about menstruation and menstrual self-care (3). Previous studies have found that girls often don't receive information about menstruation before menarche, are unprepared to manage menstrual bleeding, and are unaware of when and where to seek support (1, 4). Parents, teachers and peers may also lack information and are frequently uncomfortable discussing sexuality, reproduction, and menstruation (1, 4–6). Beyond an understanding of the physiological elements of menstrual cycle and self-care, research has highlighted that taboos and restrictions surrounding food consumption, bathing, or social participation while menstruating impact life during menses in a variety of ways (7, 8). Pain associated with menstruation impacts most girls (9). Health care services are often not sensitive to menstrual pain or problems, and the use of pharmaceutical pain relief and traditional remedies varies across contexts (1, 5, 10, 11). Further, girls report difficulties accessing sufficient and comfortable absorbents to manage menstruation, often lack access to soap and functional and secure water, sanitation and hygiene (WASH) facilities, and mechanisms for private disposal of used menstrual products (5, 11–15). Schools often do not have the WASH facilities to support adolescent girls and female teachers to manage menstruation, resulting in difficulties for female teachers to perform their teaching duties, and girls may miss school while they are menstruating (14). These challenges have been reported to contribute to school absences and participation, with potential implications for educational attainment and performance (1).

While qualitative studies have outlined the difficulties facing girls during menstruation, there is limited quantitative research estimating the extent of unmet menstrual health needs or testing the relationships between unmet needs and consequences for health and education. A study conducted in Uganda by Miiro and colleagues found that menstruation was associated with school absenteeism. Using diaries the authors found that girls reported absence more than a quarter of period days compared to only 7% of non-period days (5). A survey of girls in Indonesia found that 11.1% reported missing at least 1 day of school during their most recent menstrual period, correlated with menstrual pain, secrecy, and supportive school WASH facilities (11). Moreover, a study conducted in 2017 in Bangladesh found that 41% of girls post-menarche missed school during menstruation, with girls' attitudes, school WASH facilities and behavioral restrictions presenting risk factors for absenteeism (15).

In Myanmar, adolescents account for almost 20% of the total population (an estimated 10 million adolescents aged 10–19 years). However, evidence to understand menstrual health among adolescent girls remains limited in this setting, as does research on the linkages between menstrual experiences and school absenteeism (14). One past qualitative study highlighted poor menstrual knowledge, misconceptions about menstruation (16) and another used a survey of adolescent schoolgirls in Mawlamyine Kyun, Ayeyawaddy Region, Myanmar to report that while many girls had access to commercial sanitary products, most girls did not change these at school and inaccessibility of menstrual materials (17). In 2016, Myanmar established a budgetary allocation for a national “WASH in Schools” program, with the Ministry of Education as the lead agency, however this has not yet been delivered. In addition, international and national non-government organizations are helping to improve WASH in schools programming to complement government efforts (18). A clear understanding of girls' menstrual health challenges in Myanmar is needed to inform these programs.

The present study took a mixed methods approach aiming to describe girls' menstrual experiences and the impacts of unmet menstrual health needs among those attending Monastic schools. Specific objectives were to: (1) explore girls' experience of menstruation, identify menstrual related challenges and their impacts on school participation through qualitative interviews and focus group discussions (FGD), and (2) test the association between menstrual health needs including knowledge, attitudes and restrictions, care practices, and social support on menstrual-related school absenteeism using quantitative survey data.

Findings can help governmental and non-governmental organizations to fill critical knowledge gaps and further improve evidence-based programming and interventions to reduce school absenteeism and improve educational outcomes and the socio-economic status of girls and women.

## Methods

This mixed-methods formative research was undertaken to inform the implementation of a life skills and sexual and reproductive health (SRH) education curriculum delivered as part of the national school curriculum through monastic schools (14).

### Study setting

The study was conducted in rural and semi-rural Monastic schools in Magway Region, located in the Dry Zone of central Myanmar. Over 85% of the population from Magway region live in rural areas. Around 79% of households have access to improved water sources and 84% to improved sanitation, lower than the national average (19). Two districts were included in this study: Magway and Pakokku, covering 11 townships with a total population of 2.2 million, 400,000 of whom are aged 10–19 years (20). In 2014, there were an estimated 34 Monastic schools providing middle school education in Magway, accounting for an estimated 3,000 students (grade 5–9) (13). They provide formal education in rural areas of Myanmar where social norms are likely to be quite conservative and rigid. The teachers themselves are young and some teachers are monks or nuns. In addition to providing education to children and adolescents from surrounding communities, monastic schools also provide education to “novice” nuns and monks. These are typically children from the poorest families for whom the Monastic schools provide an education and guardianship role. Novice students board at Monastic schools, with costs covered by community donations.

### Site selection and sampling

A list of all Monastic schools in the selected districts providing education up to at least grade 7 was provided by the Ministry of Health and Sports and Ministry of Religious Affairs. Of the 26 invited schools, 16 consented to participate and could be reached during the data collection period with travel restricted due to heavy flooding.

### Participant sampling and data collection

Data were collected by trained researchers between August and December 2016. The quantitative survey and qualitative interviews were conducted concurrently; participants for the qualitative interviews were sampled from those who completed the quantitative survey.

### Quantitative cross-sectional survey

A cross-sectional survey was undertaken at each of the 16 participating schools. All students aged 11–18 attending school on the day of the survey who provided individual and parental/guardian consent participated. Of this larger sample, 421 girls who had experienced a menstrual period were asked to complete survey questions about their self-care practices, support, and menstrual-related absenteeism and were included in the present study.

Questionnaires were administered as a self-completed paper form, with a facilitator present to guide students through the questions by reading each question aloud. Major themes explored in the questionnaires were informed by the ecological framework for MHM research, developed by UNICEF and Emory University (21) and a review of the existing life skills curriculum. There were 7 key domains: Socio-demographic characteristics; Puberty and reproductive knowledge; Contraception; HIV and sexually transmitted infections; Reproductive health education at school; Menstruation and Menstrual hygiene practices; and impacts on health and wellbeing. Specific items (60 in total) were informed by established survey tools defined by UNICEF, WHO and WaterAid, and a review of the relevant literature (22). All tools were refined and adapted to the Myanmar context in consultation with in-country partners and following pre-testing with teachers, mothers and school students from non-participating school. Hard copy responses were double entered into an online version of the survey for analysis.

### Survey measures

#### Outcome

##### Absenteeism

Girls were asked to report if they had ever missed school during menstruation and the frequency with which this occurred. For binary and multivariable analysis, reporting that they had missed any days of school during the last menstrual period was used as a dichotomous outcome variable. Respondents also indicated the reasons they ever missed school.

#### Exposures (menstrual health needs)

##### Knowledge

Knowledge about the menstrual cycle was captured through correct responses to 11-items. Questions related to knowledge about menstrual anatomy, timing and links to reproduction were included such as “*Normally a girl menstruates about once every month”* and “*Pregnant women menstruate.”* Girls and boys indicated for each statement if they believed it was True, False or they did not know. The proportion of items correct (with “don't know” considered an incorrect response) was calculated as a percentage for analysis.

##### Physical symptoms

Girls indicated physical symptoms associated with menstruation through a single item which asked them to indicate if they had ever experienced any problems while menstruating including: pain that stopped you doing normal activities, feeling tired, dizzy or faint, difficulty concentrating, or heavy bleeding which was clarified as having four or more very soaked cloths or pads per day.

##### Social support and confidence managing menstruation

Girls were asked to report if they “agreed” or “disagreed” with a series of statements about their confidence to manage menstruation and seek support. Social support was captured as those who felt confident to “talk about menstruation” with various support sources such as their mother or a teacher, as well as feeling confident to “*ask a teacher for a menstrual pad or other menstrual hygiene material.”* Confidence to manage menstruation was captured by those who agreed that “*I feel confident that I could manage my menstruation if I got my period while at school.”*

##### Restrictions

This included girls' belief that it was dangerous for a girl to do physical activity, bathe, or eat certain foods during menstruation, and their agreement that girls should not cook, enter religious building, or attend school during menstruation. The number of restrictions endorsed (from 0 to 6) was used as a continuous variable.

##### Attitudes toward menstruation

Four opinions regarding menstruation were combined to represent girls' attitude toward menstruation. Girls were asked if they disagreed or agreed with statements that menstruation should be kept secret, not discussed with men, is shameful or taboo, and that menstruating girls are dirty. Attitudes toward secrecy and shame were selected as those reflecting this theme identified in the qualitative interviews and the score from 0 to 4 used as a continuous predictor of absenteeism, with higher scores representing more negative attitudes.

##### Menstrual practices

A series of questions asked girls to describe practices undertaken to manage menstrual bleeding during their most recent menstrual period. This included the types of material used, changing frequency, disposal location and laundering practices. As many girls reported traveling home to eat lunch and changing there, only the type of menstrual material used was selected as a hypothesized contributor to school absences.

#### Qualitative interviews

Five schools were purposively selected from the 16 participating schools, including two larger schools (over 300 students) and three smaller schools (<100 students). We invited participants who gave voluntary consent and met eligibility criteria for the qualitative interviews. Four FGD's were conducted at each (two with girls and two with boys), with separate FGDs for older (14–16 years) and younger (11–13 years) adolescents. A range of participatory methods were used, including activities such as body-mapping, ranking activities, and designing the ideal “girls' latrine” to explore perceptions, taboos, myths and terminologies related to menstruation, menstrual management, school absenteeism, school WASH facilities, and views on proposed interventions.

Ten in-depth interviews (IDIs) were conducted with adolescent girls who had reached menarche to further investigate the practices, impact, and challenges related to menstruation. These participants were purposively selected and included a small number of girls living with disability (*n* = 3) and novice students (novice nuns) who was willing to participate in the study and provide voluntary, informed assent and parental consent to explore in more detail the practices, determinants, impacts and challenges of menstruation and MHM. Interviews lasted 45–60 min.

Mothers with one or more adolescent aged children (aged 10–18 years) were recruited from communities near these five schools to participate in 5 FGDs that lasted 45–60 min. Topics discussed included parental roles in communicating knowledge, information, attitudes, and practices regarding menstruation, to understand parental perceptions of menstrual-related challenges that girls face in schools.

Twenty-four key informant interviews (KIIs) were conducted with teachers, members of the Regional and/or Township Supervisory Committees of Monastic Schools, basic health staff (BHS) and school health teams to examine perceptions about the provision of LSE-SRH education and identify challenges and enablers to improve menstrual experiences in school setting. Interviews lasted 30–45 min.

Interviews and FGDs were audio-recorded and transcribed into Burmese for analysis. Selected quotations were translated into English by bilingual research team members.

### Consent and ethical considerations

Permission to conduct the study was obtained from the Regional Supervisory Committee of Monastic Schools and the principal or school administrator of each participating school. Prior to the study, parents/guardians were contacted through the school head-teachers and class teachers who explained and distributed information sheets about the study and consent forms to their students to take them home and inform their parents/guardians. Participating schools had sent invitation letters to parents of enrolled students and the research team conducted community information event requesting for parental consent. To participate in the study, written assent was required from students aged under 18 years along with a parent or guardian's consent, and written consent was required from those aged 18 years or older.

The study was approved by the Department of Medical Research Ethics Review Committee (035/16) in Myanmar and the Alfred Human Ethics Committee (59/16) in Australia. All field researchers received intensive training on research ethics, including research with young people.

### Analysis

Qualitative analysis followed a framework approach. After familiarization with the Burmese transcripts, an initial coding frame was developed to capture findings across the objectives for the broader formative study (including: sexual and reproductive health knowledge and attitudes, life skills education, menstrual practices and determinants, and impacts of menstruation) and more granular themes and subthemes (total 89 subcodes). Four researchers based in Myanmar then coded transcripts line by line, regularly reviewing the coding framework, and provided English summaries and quotations for each interview and focus group across each theme in the coding framework. For the present study, NOM reviewed the English framework summary for topics related to menstrual experiences and impacts and developed revised themes capturing reported menstrual health needs and consequences for girls' lives, in consultation with JH and a member of the original qualitative team (ZYS).

Quantitative analysis was undertaken in Stata 16. Descriptive analyses were used to describe menstrual health needs and consequences for girls' lives identified through the qualitative portion of the work. To explore the contribution of menstrual health needs to school absenteeism survey questions reflecting key menstrual health needs identified through the qualitative work and past research were selected. Binary logistic regressions were used to describe the individual relationships between these menstrual health needs (exposures) and school absence due to menstruation. Binary relationships (*p* < 0.10) were then included in a multivariable logistic regression to test their relationships.

Survey questions were selected to capture the themes developed through the qualitative work along with those identified in past research. Binary and multivariable logistic regressions were used to describe the relationship between menstrual health needs and girls' self-reported school absence due to menstruation.

## Results

### Respondent characteristics

A total of 298 individuals participated in qualitative activities. This included: 10 FGDs with girls, 10 FGDs with boys and 5 FGDs with mothers; 10 IDIs with girls; and 24 KIIs with relevant stakeholders.

A total of 421 girls who had ever experienced a menstrual period participated in the quantitative survey and responded to questions about their experience of menstruation and thus were included in the present study. This was a subset of the total 1,431 students (girls and boys) who had participated in the cross-sectional survey. In the sample of post-menarche girls used for this study, the mean age was 14 years (*SD* 1.46) and ages ranged from 11 to 22 years. Approximately 77% of the post-menarche girls were in Grade 7–9, with smaller proportions from Grades 5, 6 and 10. Half the sample were from Magway Township monastic school (49.9%) and half from Pakokku Township monastic schools (50.1%). Of the student sample, 15% boarded at school and 18% were novice monks or nuns.

This results section presents the themes describing menstrual experiences and consequences for girls' lives, accompanied by quantitative description relevant to each theme. This is followed by bivariate and multivariable analysis of the relationships between menstrual health needs and school absenteeism.

### Knowledge and information provision

In FGDs, many girls reported that they felt unprepared for menarche and experienced fear and anxiety in response to their first period. Menarche prompted girls to talk to their mothers, sisters, friends, or female teachers to receive more information or advice. Some mothers expressed the view that girls should not receive information about menstruation or reproduction prior to menarche, fearing it would frighten them or that they themselves did not feel comfortable discussing menstruation with their daughters. A minority of girls echoed this sentiment, while others expressed that they would have liked information earlier and wanted more detailed information about menstruation and menstrual management.

“*Tell them only after menarche, Mothers worry that their daughters will get shy if they are told in advance*.” [FGD-5P-Mothers]

Girls and boys both reported wanting to learn about menstruation and reproductive health at school in separate groups with a male and female teacher, respectively. Some students felt that education about menstruation should be only given to girls, with boys feeling that menstruation was not relevant to them and girls concerned that boys having greater knowledge may result in increased teasing.

“*We don't want the boys to know we are having menstruation. The boys need not know about menstruation. Girls need to know everything about this. Father should not know too. We will suffer from sin. Boys need not know about menarche. Because they will tease and pester us.”* [FGD-4F-Girls]

Most girls participating in the quantitative survey reported being aware of menstruation prior to their first period 84% (354/421). Results on the 11-item knowledge test, however, showed many deficits in understanding the biology of menstruation and its links to reproduction, scoring only a mean of 57%.

### Support from others

Girls identified mothers and sisters as sources of information and practical support for menstruation, including providing access to menstrual materials. While some felt embarrassed asking mothers for help during menstruation, most felt comfortable seeking this support.

“*Mother told me to tell her when I have my menses and that she will help me. Mothers buy the pad for me. She would do everything to help me. If there is pain, she would give medicine. If I feel sick, she would give [traditional] medicine.”* [FGD-3F-Girls]

Female friends were also identified as sources of support, providing information, buying or sharing menstrual materials such as commercial pads, or supporting menstruating girls to undertake daily tasks. Female teachers were a source of support for some, providing access to menstrual materials or assisting girls in hiding soiled clothes from males.

“*If they know a girl is having her menstruation, the people around will take care. Like my friend - when the teacher asks her to go and bring something, friends around her would help to bring it. They don't leave her out - they treat her just like before. Even better than before.”* [FGD-4L-Girls]

Consistent with the qualitative findings, most girls (91%) reported feeling confident to discuss menstruation with their mother or female relative, and 76% to discuss with a female friend. Only 41% were confident discussing menstruation with a female teacher, with 34% feeling confident they could ask a teacher for a menstrual pad if needed. Very few girls were confident to talk to their father (3%), a male teacher (2%), or boys (1%) about their period.

Adolescent girls in the FGDs highlighted a range of misconceptions, behavioral restrictions and expected practices surrounding menstruation. Many girls, boys, and mothers reported that being close to boys during menstruation placed girls at risk of pregnancy.

“*During the period, girls should not go to crowded places and be near to boys. If they go, they will get pregnant. If a boy touches the longyi of the girl who has her period, the girl can get pregnant”* [FGD-4L-Girls]

Other taboos included not washing hair whilst menstruating, not eating spicy or sour foods, not drinking cold drinks, not eating pickled tea, not washing your feet with already used water, not handling cow dung and not sitting under any trees with “nat” (spirits). Dietary restrictions were often linked to concerns that they would affect menstrual bleeding, although not all girls knew the reasons for various restrictions. Participants also reported that menstruating girls were restricted from participating in religious activities or entering holy places, and were expected to take on fewer household chores and activities. Restrictions and taboos were typically learned from, and reinforced by, parents (particularly mothers), senior relatives or friends.

“*When menstruating, you should not wash your hair; you must not drink cold drinks as your bleeding will clot. Pickled tea leaves will make menstruation stop. You must not eat sour things, will make heavy flow of blood and you can die.”* [FGD-3F-Girls]

In the quantitative survey, many post-menarche girls agreed that menstruating girls should not be allowed to cook or prepare food (38%), enter religious buildings (50%) or attend school (12%).

### Secrecy and shame

Most girls described menstruation as something shameful, reporting feeling dirty and not wanting to let others know that they were menstruating. This shame and secrecy resulted in some girls avoiding school or participating in school or other activities to avoid others discovering their menstrual status or because they regarded themselves as dirty and feared that others could smell their menstruation.

“*Mostly, I don't attend school, as I don't want others to know I am having my period.”* [FGD-1H-Girls]

Girls felt it was particularly important to hide menstruation from boys, reporting that boys at school would tease them if they knew they were menstruating or stained their longyi. Teachers and health workers interviewed also expressed being aware of this teasing.

“*I was only in 6th standard and did not know anything. There was a big stain on my longyi. When the teacher saw and called me, only then I knew. I had to change my longyi. Then the boys found out and sang a song that I have the “weather” (menstruation) teasing me*. [FGD-4F-Girls]

In FGDs, boys expressed negative views of menstruation as dirty.

“*Menstruating is not good. It is unclean I think… when menstruation comes, there is smell, so people will not want to be near that person.”* [FGD-1L-Boys]

In the quantitative survey, 48% of girls agreed that menstruation should be “kept secret,” 32% that it was shameful or taboo to discuss menstruation, and 33% agreed that menstruating girls are “dirty.” Almost all girls (91%), agreed that menstruation was women's business and shouldn't be discussed with men.

### Physical symptoms

Menstrual pain was reported by many girls and resulted in school absenteeism and difficulties for girls. Heavy bleeding and dizziness were also described, with some girls associating feelings of nausea and dizziness with heavy bleeding. Girls described using traditional medicines such as *ShanPyoMai, Kaythipan*, and *ThwayHsay* to manage pain and blood flow, with fewer girls reporting use of analgesics. Only a few participants mentioned using vitamins or supplements during their period. Some described taking boiled hot water and salt for the pain relief or eating certain foods to regulate menstruation. In individual interviews many girls noted mood changes during their period, such as feeling irritable, sad, or unable to concentrate.

“*To avoid menstruating, I would eat spicy Laphet salad (pickled green tea salad)”* [FGD-4F-Girls]

In the quantitative survey 29% of girls reported pain that stopped them from doing regular activities while 22% reported feeling tired and 13% dizzy or faint during their period.

### Managing menstrual bleeding at school

Most girls reported using disposable pads to manage menstrual bleeding. These were the preferred material and described as affordable (costing 500–1,000 Myanmar Kyat, US $0.40–0.70). In absence of pads, and more often at home, girls used 2–3 layers of *longyi*, or cloth as menstrual absorbent. In the quantitative survey, 89% of the sample reported mainly using sanitary pads, 2% longyi, 3% cloth, and 5% underwear alone.

“*In this village, most of the girls use Eva [a common brand name used to refer to all brands of pads]. They also wear black longyi. Sometimes, when we go to social events we would use 2 pads together. Panties and longyis are folded 4 to 5 layers and worn inside the panties. After this, we wear black color longyi”* [FGD-4F-Girls]

Adolescent girls, mothers, and teachers all stated that purchasing menstrual materials from stores in the villages or near the school was easy. However, some girls felt too shy to buy materials or embarrassed to bring them to school. Most girls commented that they were unable to get menstrual materials at school but noted that they could ask friends or teachers to cover stains on their longyi and travel home at lunch time to change clothes and collect materials. Participants felt that schools should have menstrual pads and analgesics available for emergencies.

“*At first, I dare not buy. Now I buy it from a shop outside the school. Last year, I brought a pad in my school bag. But I thought it can send me to hell, and so I don't bring it this year. I also worry that boys may see it if I keeps it in the pouch beside my bag.”* [IDI-1F-Girls]

Girls noted that if their longyis got stained by menstrual blood, they would turn it around to hide the stain and went home. Dark colored longyi were preferred during menstruation as stains were less visible. In one school, girls noted that their green colored uniform meant stains were more visible and a challenge to conceal.

School WASH facilities often lacked privacy, with girls and boys toilets in close proximity, and many cubicles lacking doors that could be locked from the inside. Girls and boys both reported that latrines were often dirty or smelly and that teachers limited the number of students or times they could visit the latrine. Girls feared revealing their menstrual status and subsequent teasing from boys when using latrines or washing their hands. They also reported in IDIs that there was nowhere to dispose of menstrual pads.

“*I am afraid also when I have to dispose pads at school, because there are many people around. I am afraid boys will see it when I dispose [of] it.”* [FGD-4K-Girls]

The limitations of school WASH facilities meant many girls traveled home to change menstrual materials, with only 37% reporting changing their menstrual materials at school during their last period. However, many students returned home for lunch, and so were able to change their materials at home during the day.

To be able to manage their menstruation comfortably at the school, girls illustrated their ideal latrines which included a door that could be locked from the inside, proper lighting, cleanliness, a rubbish bin with a lid for disposal of pads, water and a place to wash their hands, a secure changing room and slippers.

*I think sanitary latrine is important for girls who are having periods. It is to wash and clean. To wash the residuals from menstruation and dirty toilet if needed* [FGD-3H-Girls]

### Consequences for school attendance and social participation

Challenges experienced during menstruation described in the themes above resulted in consequences for girls' education and social participation. Menstrual pain and symptoms made it challenging for girls to attend school. Behavioral restrictions on entering holy places or participating in religious practices meant some girls avoided school as these activities were mandatory activities of the school day, while others sought to avoid being around other people, particularly boys and male teachers, during menstruation.

“*Sometimes, she is absent from school because she did not want to enter into the shrine room to change flowers. She thought it can cause her to go to hell. She doesn't want to be absent as she is afraid of missing classes and lessons.”* [IDI-1E-Girls]

Embarrassment and shame surrounding menstruation, and fear of staining and teasing all meant many girls avoided school or participated less is class activities when they did attend. Some students commented that if their period started whilst they were at school, they would ask permission from the teacher and return home. They noted that they could not concentrate on studies due to the fear of staining their clothes and avoided standing in front of the class to conceal their menstruation from others.

“*My friends would miss school for four days whenever they have menses. They would stay in their rooms only. They won't eat food too. They stay in the dormitory near the school. Because they feel shy, they won't come to school”* [FGD-4F-Girls]

Teasing from boys was a common fear, and particularly distressing. Restrictions meant that some girls avoided social settings or undertaking certain household chores during menstruation. Although not explicitly linked to absenteeism in qualitative interviews and FGDs, unsupportive school sanitation infrastructure and the need to travel home to access and change menstrual materials were identified as important challenges for girls.

### Associations between menstrual health needs and school Absenteeism

Table 1 reports the proportion of girls reporting school absences due to menstruation, reasons for absence, and consequences for concentration.

**Table 1:** Proportion of post-menarche girls reporting school difficulties due to menstruation (*n* = 421).

**Consequences for school**	**%**	***N***
Missed school due to the last menstrual period	12.83	54
**Frequency of missing school due to menstruation**
Average days of missing school during last menstrual period	1.5 days	
Never missed school due to menstruation	85.75	361
Miss school less than every second period	2.61	11
Miss school during approximately every second period	9.74	41
Miss school every period	1.90	8
**Reasons for missing school due to menstruation (*****N*** **=** **60)**		
Afraid of staining clothes	33.33	20
Afraid of smelling bad	10.00	6
Afraid of others making fun of her	6.67	4
Blood flow is too heavy	11.67	7
Gets too much pain	28.33	17
Feels too tired	3.33	2
There isn't anywhere private at school to wash or change	5.00	3
Religious or cultural reasons prevent her from attending school	1.67	1
**Participation at school**		
Ever had trouble concentrating during menstruation	18.53	78

A total 12.8% of girls reported missing school due to their last menstrual period and an average 1.5 days of school were missed due to menstruation (see Table 1). Amongst the 60 girls who reported missing days of schools, 6 (10%) missed half days, 37 (61.67%) missed 1 day, 8(13.33) missed 2 days, 7 (11.67) missed 3 days, 1 (1.67) missed 5 days, and 1 (1.67) missed 6 days. Table 2 describes the relationships between menstrual health needs and absenteeism, with challenges including: physical menstrual symptoms, knowledge, restrictions, secrecy and shame, support from others, confidence, and blood management. Girls' odds of school absenteeism decreased with each increasing grade level. Experiencing significant menstrual pain or heavy bleeding was associated with increased odds of absenteeism in binary and multivariable comparisons. Knowledge about menstrual biology and the menstrual cycle was not associated with school absenteeism.

**Table 2:** Bivariate and multivariable associations between menstrual health challenges and menstrual-related school absenteeism.

	**Total *N* (%)**	**0 days missed**	**1 or more days missed**	**OR (95%CI)**	**aOR (95%CI)**
**Grade level**					
Grade level (cont.)				0.77 (0.62–0.97), *p* = 0.025	0.76 (0.60–0.97), *p* = 0.031
**Physical symptoms**					
Experience significant pain	122 (28.98)	100 (27.25)	22 (40.74)	1.84 (1.02–3.31), *p* = 0.043	2.10 (1.10–4.00), *p* = 0.023
Heavy bleeding	41 (9.74)	29 (7.90)	12 (22.22)	3.33 (1.58–7.02), *p* = 0.002	3.33 (1.51–7.34), *p* = 0.003
**Knowledge**					
Menstrual knowledge (% score)	56.57 (17.49)	56.50 (17.40)	57.07 (18.21)	1.00 (0.98–1.02), *p* = 0.823	-
**Restrictions**					
Endorsement of restrictions and taboos (0–6) (M, SD)	3.67 (1.31)	3.62 (1.32)	4.02 (1.16)	1.27 (1.01–1.60), *p* = 0.037	1.14 (0.89–1.48), *p* = 0.309
**Secrecy and shame**					
Negative attitudes toward menstruation (0–4) (M, SD)	2.50 (1.10)	2.45 (1.09)	2.83 (1.11)	1.39 (1.06–1.83), *p* = 0.017	1.34 (0.99–1.80), *p* = 0.053
**Support from others**					
Confident to talk to female relative about menstruation	383 (90.97)	333 (90.74)	50 (92.59)	1.28 (0.43– 3.75), *p* = 0.657	-
Confident to talk to friends about menstruation	318 (75.53)	277 (75.48)	41 (75.93)	1.02 (0.53–2.00), *p* = 0.943	-
Confident to talk to female teacher about menstruation	172 (40.86)	148 (40.33)	24 (44.44)	1.18 (0.67–2.10), *p* = 0.566	-
Confident to ask a teacher for a pad	144 (34.20)	119 (32.43)	25 (46.30)	1.80 (1.01–3.20), *p* = 0.047	1.93 (1.06–3.54), *p* = 0.031
**Confidence**					
Confident to manage menstruation at school	322 (76.48)	283 (77.11)	39 (72.22)	0.77 (0.41–1.47), *p* = 0.430	-
**Menstrual management**					
Menstrual material used during last period					
Disposable pad	374 (88.84)	329 (89.65)	45 (83.33)	0.58 (0.26–1.27), *p* = 0.173	-
Improvised material (e.g., cloth, longyi, underwear, cotton wool)	47 (11.16)	38 (10.35)	9 (16.67)	Ref	-

Greater endorsement of restrictions surrounding menstruation was associated with significantly higher odds of school absence in the binary comparison, with those not absent endorsing a mean 3.6 restrictions and those missing school endorsing a mean 4.0 restrictions. However, this was no longer statistically significant in the multivariable comparison. Girls' attitudes toward menstruation as something that was shameful and should be kept secret were also associated with increased odds of absenteeism in the binary comparison, with those not absent endorsing 2.5 attitude items compared to a mean 2.8 among those who missed school. This was also no longer statistically significant in multivariable comparison, although trended toward significance with each additional attitude item associated with a 1.34 increase in odds of school absenteeism (95%CI 0.99–1.80).

Girls' confidence to discuss menstruation with a female relative, friend, or teacher was not associated with school absenteeism. However, confidence to ask a teacher for a pad was associated with greater absenteeism in binary and multivariable analysis.

A total 77% of girls who did not miss school reported being confident to manage their menstruation at school, compared to 72% of those who were absent, but this difference was not statistically significant.

The type of menstrual material used as absorbent during the last period was also not associated with absenteeism.

## Discussion

This is the first study to comprehensively explore menstrual health amongst adolescent girls in Myanmar and its related outcomes. Qualitative inquiry explored girls' experiences of menstruation, identified menstrual related challenges, and the impact on their lives. This was complemented by quantitative survey data that measured the prevalence of menstrual needs and tested the association between menstrual health needs and menstrual-related school absenteeism.

Qualitative data revealed a range of menstrual health challenges for girls including access to knowledge and social support, restrictive expectations, stigma surrounding menstruation, difficulties managing menstrual bleeding such as accessing sufficient menstrual products and supportive school infrastructure, along with menstrual pain. These were consistent with past research in LMIC settings, and the integrated model of menstrual experience which recently synthesized these findings (1). Girls described fear and distress associated with menstruation and consequences for school attendance and participation. In the quantitative survey, we found that 12.8% of girls missed schools due to their last menstruation, missing on average 1.5 days of school. This is lower than the studies from Indonesia, Bangladesh and Africa contexts but is still a substantial amount of missed education (5, 11, 15, 23). Girls in lower grades were more likely to report absenteeism due to menstruation, perhaps suggesting those recently starting their period struggled more or representing selective attrition.

Many girls reported that they felt unprepared for menarche and would like earlier and more comprehensive information about menstruation. In Myanmar, Life Skills Education curriculum covers a broad range of topics related to sexual and reproductive health (SRH), including menstruation. Our findings suggest that current education curriculum and implementation should be adapted to better address menstrual health knowledge gaps. Students in focus group discussions stated a preference for sex-separated classes with a same-sex teacher. Although mothers are the preferred source of menstrual information for girls, many mothers felt ill-equipped to provide advice and support to their daughters and were concerned this information may scare them. Mothers and community members also communicated restrictions and taboos around menstruation which may negatively impact girls. Informational support to mothers could help them to discuss these issues with their daughters and dispel fears (3, 24, 25).

Beyond knowledge about menstruation, attitudes that menstruation is dirty or unclean and should be kept secret, along with taboos restricting girls' behavior during menstruation, were highlighted through our study. These contributed to significant challenges for girls' wellbeing and social engagement during menstruation. Fear of teasing by boys, results in girls being reluctant to bring sanitary pads to schools and anxiety about using school WASH facilities to change menstrual products. In our quantitative analysis, negative attitudes toward menstruation were associated with menstruation-related absenteeism in binary comparisons. This contrasts with limited biological menstrual knowledge which was not associated with absenteeism. Findings suggest that dismantling shame and fear surrounding menstruation, and working with communities to address negative attitudes and taboos may help reduce menstrual related absenteeism and support girls during menstruation.

Interview and focus groups noted that WASH facilities were often dirty and lacked privacy, with girls' and boys' toilets in close proximity. Girls reported significant fear of teasing by boys when using latrines or washing hands. Moreover, many girls reported that there was nowhere to dispose of menstrual pads as no schools had bins for disposal of menstrual materials. Questions to explore the role of WASH facilities in the quantitative analyses were limited and many girls reported going home at lunch which facilitated changing menstrual materials outside of school WASH facilities.

Social support was emphasized in girls' experiences in the qualitative investigation, however girls' confidence to discuss menstruation with a female relative, friend or teacher was not associated with school absenteeism. However, confidence to ask a teacher for a pad was associated with greater absenteeism in binary and multivariable analysis. One plausible explanation for this surprising finding is that girls lacking sufficient access to pads and have experience asking teachers for pads and so feel confident doing so. This may suggest that girls struggling to access enough menstrual materials was a driver of school absenteeism but was poorly captured by our question which asked about the type of product use. Most girls reported using pads and this was not associated with absenteeism in quantitative analysis. Questions to capture unmet needs more clearly, such as the Menstrual Practice Needs Scale could explore this effect in future studies (26). Alternatively, this effect may have been observed due to unmeasured confounders.

Menstrual pain was an important challenge reported by many girls in qualitative interviews, and pain and heavy bleeding were associated with significantly higher levels of absenteeism in our multivariable comparison. This is consistent with studies with adolescent girls in Indonesia, Bangladesh, and Uganda (5, 11, 15). Addressing pain and other physical menstrual symptoms is essential for comprehensive menstrual health (27) and to address menstrual related absenteeism (24).

### Study strengths and limitations

Our mixed methods investigation sought to understand the perspectives of girls, boys, parents, and teachers and provide a nuanced understanding of girls' experiences of menstruation in monastic schools in Myanmar. Findings informed our quantitative analysis to test one dimension of the qualitative results; the unmet menstrual health needs that may contribute to school absenteeism and triangulate conclusions drawn from the qualitative findings. We note that this was an exploratory study and study areas were selected purposively to represent diverse cultural, socioeconomic, and geographical settings. The findings may not represent all monastic schools either regionally or nationally. We utilized self-reported data on school absenteeism, this exposure variable may be underestimated or overestimated. Although a small number of interviews were undertaken with students with disabilities, this number was insufficient for more detailed analysis on the needs of this population.

School absenteeism was assessed as full days missed, but emerging evidence suggests that girls often miss hours or half days of school, which is not captured in our data. Assessing the quality of school participation and performance as outcomes may have strengthened our study and permitted a more nuanced understanding of the impact of unmet menstrual needs on educational outcomes, beyond absenteeism. Additionally, there is a need to assess the impacts on other outcomes such as psychosocial wellbeing and mental health (28, 29), which is often overlooked. The predictors of these outcomes may be different to those for absenteeism.

## Conclusions

Our findings, critical to informing targeted approaches in Myanmar, contribute to the growing evidence that adolescent girls face substantial challenges during menstruation, adversely impacting on their education and wellbeing. Providing age appropriate education and addressing the shame and taboos currently associated with menstruation are important components of a comprehensive menstrual health response. In addition, our study highlights the need to ensure access to menstrual resources and WASH facilities, along with access to adequate menstrual pain relief.

## Data availability statement

The raw data supporting the conclusions of this article will be made available by the authors, without undue reservation.

## Ethics statement

The studies involving human participants were reviewed and approved by the study was approved by the Department of Medical Research Ethics Review Committee (035/16) in Myanmar and the Alfred Human Ethics Committee (59/16) in Australia. All field researchers received intensive training on research ethics, including research with young people. Written informed consent to participate in this study was provided by the participants' legal guardian/next of kin.

## Author contributions

ZS, NM, KT, JD, LB, EK, PA, and JH: conceptualization and methodology. ZS, NM, and JH: formal qualitative analysis and formal quantitative analysis. ZS, JD, LB, EK, and PA: investigation. PA, EK, and JD: resources. ZS and NM: writing—original draft. JH, KT, JD, LB, EK, and PA: writing—review and editing. JH and JD: supervision. Authors have approved the final manuscript.

## Funding

The analysis presented in this manuscript was supported by a NHMRC Ideas Grant (GNT2004222). PA was supported by a NHMRC Early Career Fellowship (#1145228).

The primary data collection and formative study were funded through the Australian NGO Cooperation Program funded by the Australian Department of Foreign Affairs and Trade, and Water AID Australia.

The authors gratefully acknowledge the contribution to this work of the Victorian Operational Infrastructure Support Program received by the Burnet Institute.

## Conflict of interest

The authors declare that the research was conducted in the absence of any commercial or financial relationships that could be construed as a potential conflict of interest.

## Publisher's note

All claims expressed in this article are solely those of the authors and do not necessarily represent those of their affiliated organizations, or those of the publisher, the editors and the reviewers. Any product that may be evaluated in this article, or claim that may be made by its manufacturer, is not guaranteed or endorsed by the publisher.
