# Supplementary material for: Adolescent girls' experiences of menstruation and schooling in monastic schools in Magway Region, Myanmar: A mixed-methods exploration
Source: Front Reprod Health. 2022 Aug 1;4:893266. doi: 10.3389/frph.2022.893266 (PMC9580682; doi:10.3389/frph.2022.893266)
Supplement: Supplementary file 1 [file Data_Sheet_1.pdf]

APPENDIX

ADOLESCENT REPRODUCTIVE HEALTH IN MYANMAR: FORMATIVE RESEARCH TO SUPPORT THE DELIVERY OF LIFE-SKILLS EDUCATION AND HYGIENE IMPROVEMENTS THROUGH MONASTIC SCHOOLS IN MAGWAY

QUESTIONNAIRE FOR GIRLS AND BOYS

FACILITATOR TO COMPLETE:

Name of researcher:		Date (dd/mm/yy):	
Start time:		End time:	
Name of school:		District:	
School ID:		Township:	

Written informed consent obtained (Please cross ✗)?

YES	
NO	

Parent /guardian consent obtained?

YES	
NO	

Has the questionnaire been completed?

YES	22
NO	23

If no, please check the missing responses with the student

Instructions to the student:

Thank you for taking the time to complete this questionnaire. This questionnaire is part of a study being conducted in schools in Magway to understand young people's knowledge and opinions about health. We are particularly interested in health issues that are important to young people your age, such as puberty, menstruation and reproductive health. The questions will ask about your knowledge about reproductive health issues, where you currently get information from, and life-skills education taught in school. Your responses are very important to us and will help to improve the information that is provided to young people in school.

The questionnaire is anonymous, which means that all your responses will be kept strictly confidential and your name will not be recorded anywhere, so no-one will know how you answered the questions. The questionnaire will not be shared with your school or teachers.

Please carefully read and answer the questions below as truthfully as you can. Please write your answer or put a cross ✗ ☐in the box next to the response that best applies to you.

If you are unsure about any of the questions please raise your hand and ask the researcher to explain.

When you are finished please hand the questionnaire to the researcher. They will check that all the questions have been answered and check with you if there is anything missing.

SECTION 1: Socio-demographic characteristics

1. How old are you?
- (Please cross ✗)

11	
12	
13	
14	
15	
16	
17	
18	

Other age (please write):

____

2. What gender are you?

Male	
Female	

3. What grade are you in at school?

Grade 5	
Grade 6	
Grade 7	
Grade 8	
Grade 9	
Grade 10	

Are you a Novice student?

Yes	
No	

Are you boarder student?

Yes	
No	

4. What religion do you identify with?

Buddhism	
Hinduism	
Catholicism	
Baptist	
Muslim	
No religion	
Other religion (please describe):	

	No difficulty	Some difficulty	A lot of difficulty	Cannot do at all
5. Do you have difficulty walking or climbing stairs?				
Do you have difficulty seeing, even if wearing glasses?				
Do you have difficulty hearing, even if using a hearing aid?				
Do you have difficulty remembering or concentrating?				
Do you have difficulty with self-care, such as washing all over or dressing?				
Do you have difficulty communicating, for example understanding or being understood?				

SECTION 2

Puberty and reproduction

6. When boys and girls begin to change from a child to an adult, known as **puberty**, they experience some physical changes. Please read the statements below and indicate with a cross ✗ whether you think each one is true, false, or whether you don't know. (Please answer all questions)

When a boy goes through puberty, he…	True	False	Don't Know
develops muscles			
has a change in voice			
grows hair on his face and body			
• has an increase in sexual arousal			
• has wet dreams			
**When a girl goes through puberty, she…**	**True**	**False**	**Don't Know**
• grows pubic and underarm hair			
• experiences growth of her breasts			
• experiences growth of her hips			
• has an increase in sexual arousal			
• starts to menstruate			

7. Below are some other statements on **reproduction**. Please indicate with a cross ✗ whether you think the statement is true, false, or whether you don't know. (Please answer all questions)

	**TRUE**	**FALSE**	**DON'T KNOW**
Both men and women can make sperm			
Eggs are released by a woman's ovaries			
An egg must be fertilised by sperm for pregnancy to occur			
A woman can get pregnant the very first time that she has sexual intercourse with a man			
There is a chance that a woman can get pregnant every time she has sexual intercourse with a man			
A woman is most likely to get pregnant if she has sexual intercourse half way between her menstrual periods			
After puberty, a man's body will continue to make sperm for the rest of his life			
Masturbation causes serious damage to health			

8. Young people have various views about relationships. Some of those views are listed below. For each one, please indicate with a cross ✗ whether you agree or disagree. (Please answer all questions)

	**Agree**	**Disagree**	**Not sure**

I believe it's all right for unmarried boys and girls to have dates			
I believe it's all right for unmarried boys and girls to hold hands			
I believe it’s all right for unmarried boys and girls to kiss			
I believe there is nothing wrong with unmarried boys and girls having sexual intercourse if they love each other			
I believe that sometimes a boy has to force a girl to have sex if he loves her			
I believe that a boy will not respect a girl who agrees to have sex with him			
I believe that most girls who have sex before marriage regret it afterwards			
I believe that most boys who have sex before marriage regret it afterwards			
I believe that a boy and a girl should have sex before they get married to see whether they are suited to each other			
I believe that girls should remain virgins until they marry.			
I believe that boys should remain virgins until they marry.			
I believe that it is sometimes justifiable for a boy to hit his girlfriend.			
I believe it's all right for unmarried people to have sex provided that			

they use a method to prevent pregnancy.			

•

• 9. Have you ever received information about **puberty and reproduction**?	Yes ☐ No ☐ → **GO TO QUESTION 11**
10. Where or from whom did you **mostly** get this information? (please put a cross ✗ for **three** of the options below)	
School teacher	
Mother	
Father	
Brother	
Sister	
Other family members	
Friends	
Health worker (nurse or doctor)	
Books/magazines/pamphlets	
Films/Videos/TV channels	
Internet	
Other (specify)______________	
11. From whom or where, would you **most** prefer to receive information about puberty and reproduction? (please put a cross ✗ for **one** of the options below)	

School teacher	
Mother	
Father	
Brother	
Sister	
Other family members	
Friends	
Health worker (nurse or doctor)	
Books/magazines/pamphlets	
Films/Videos/TV Channels	
Internet	
Other (specify)______________	

12. Have you ever received information about **relationships -** I mean how boys and girls should treat each other? (Please select one and mark X.)	Yes ☐<br>No ☐ → **GO TO QUESTION 14**
13. Where or from whom did you **mostly** get this information? (please put a cross ✗ for **<u>three</u>** of the options below)	
School teacher	
Mother	
Father	
Brother	

Sister	
Other family members	
Friends	
Health worker (nurse or doctor)	
Books/magazines/pamphlets	
Films/Videos/TV channels	
Internet	
Other (specify)_____________	
14. From whom, or where, would you **most** prefer to receive information about relationships? (please put a cross ✗ for **one** of the options below)	
School teacher	
Mother	
Father	
Brother	
Sister	
Other family members	
Friends	
Health worker (nurse or doctor)	
Books/magazines/pamphlets	
Films/Videos/TV channels	
Internet	

Other (specify)________________	

SECTION 3

Contraception

15. Below is a list of different methods people can use to prevent pregnancy. For each one, please indicate with a cross ✗ whether you have never heard of the method, have heard of it but don't know how to use it, or have heard of it and know how to use it. (Please answer all questions)

	**Never heard of**	**Heard of but don't know how to use**	**Heard of and know how to use**
Condom			
Oral contraceptive pill			
Emergency contraception			
Injectable contraception			
Contraceptive implant			
Withdrawal			
Intrauterine device			
Tubal ligation			
Vasectomy			

16. Below are some statements about various ways or methods that people can use to prevent pregnancy. For each one, please indicate whether you

believe it to be true, false, or you don't know by placing a cross ✗ in the appropriate box. (Please answer all questions)			
	**True**	**False**	**Don't Know**
Women can have an operation to stop them from having any more children			
There is no operation for men to stop them from having any more children			
Women can take a pill every day to prevent pregnancy			
Women can have an injections by a health provider every 3 months to prevent pregnancy			
Men can have an injections by a health provider every 3 months to prevent pregnancy			
Using a condom during sexual intercourse can prevent pregnancy			
One male condom can be used more than once			

17. People have different opinions about contraception and condoms. For each of the opinions below please indicate using a cross ✗ whether you agree or disagree. (Please answer all questions)

	**Agree**	**Disagree**	**Don't know**
I believe contraception should only be used by married people			

I believe contraception should only be used by couples who already have children			
I believe contraception encourages young people to have sex			
I believe it is the woman's responsibility to prevent pregnancy			
I believe it is important for young people to receive information about contraception			
I believe it is the man's responsibility to carry condoms			
I believe condoms reduce pleasure during sex			
I believe a girl can suggest to her partner that he use a condom			
I believe that a boy can suggest to his partner that he use a condom			
I believe condoms only need to be used with casual partners			
I believe that if a girl suggests using a condoms to her partner it means that she doesn't trust him			
I believe that condoms encourage young people to have sex			
I believe that unmarried people should use condoms			

18. Have you ever received information or education about **contraception and condoms**?<br>(Please select one and mark X)	Yes ☐<br>No ☐ → **GO TO QUESTION 20**
19. Where or from whom did you **mostly** get this information? (please put a cross ✗ for **<u>three</u>** of the options below)	
School teacher	
Mother	
Father	
Brother	
Sister	
Other family members	
Friends	
Health worker (nurse or doctor)	
Books/magazines/Pamphlets	
Films/Videos/TV channels	
Internet	
Other (specify)______________	
20. From whom, or where, would you **most** prefer to have received information about contraception and condoms? (please put a cross ✗ for **<u>one</u>** of the options below)	
School teacher	
Mother	
Father	
Brother	

Sister	
Other family members	
Friends	
Health worker (nurse or doctor)	
Books/magazines/Pamphlets	
Films/Videos/TV channels	
Internet	
Other (specify)_______________	

SECTION 4

HIV and sexually transmitted infections

21. Have you heard of HIV or AIDS (use local terms)?<br>• (Please select one and mark X.)	Yes ☐<br>No ☐ →	GO TO QUESTION 23

22. Below are some statements about HIV/AIDS. Please indicate with a cross ✗ whether you think the statement is true, or false, or whether you don't know. (Please answer all questions)

	True	False	Don't know
It is possible to cure AIDS			
People can take a simple blood test to find out whether they have HIV			
People can get HIV from mosquito bites			
People can get HIV from sharing food with a person who has HIV			
People can get HIV because of shaking hands with a person who has			
It is possible for a healthy-looking person to have HIV			
It is possible for a mother to transmit HIV to her baby during pregnancy			
It is possible for a mother to transmit HIV to her baby during childbirth			

HIV **cannot** be transmitted in breast milk			
Using a condom during sex can reduce the chance of a person getting HIV			
Abstinence (not having sex) is one way to avoid getting HIV			
A person can reduce their chance of getting HIV if they only have one uninfected sexual partner who has no other sexual partners			

23. Below are some statements about sexually transmitted infections (STIs). Please indicate with a cross ✗ whether you think the statement is true, or false, or whether you don't know. (Please answer all questions)

	**True**	**False**	**Don't Know**
STIs can be spread during vaginal sex			
STIs can be spread during anal sex			
STIs can be spread during oral sex			
You can get an STI from a toilet seat			
You can always tell if someone has an STI by looking at them			
If an STI is untreated it can cause infertility			
Using a condom during sex can reduce the chance of a person getting an STI			
Condoms are the only form of contraception that can prevent STIs and pregnancy			

STIs nearly always go away on their own without treatment			
It is possible for someone with an STI to have no symptoms			
Discharge from the penis can be a sign of an STI in men			
Discharge from the vagina can be a sign of an STI in women			
It is possible to get an STI even if a person only has sex once			

24. People have different opinions about HIV and STIs. For each of the opinions below please indicate using a cross ✗ whether you agree or disagree. (Please answer all questions)

	**Agree**	**Disagree**	**Not Sure**
If a member of my family had HIV I would want it to remain a secret			
If a member of my family became sick with AIDS I would be willing to care for them			
I would buy fresh vegetables from a shopkeeper if I knew they had HIV			
I believe that if a student has HIV but is not sick they should not be allowed to continue attending school			
I believe that if a teacher has HIV but is not sick they should not be allowed to continue teaching at the school			

I would stop spending time with a friend if I knew they had HIV			
I believe that only people who have many sexual partners get STIs			

25. Have you ever received information about **HIV and STIs**<br>(Please select one and mark X)	Yes ☐<br>No ☐ → GO TO QUESTION 27
26. Where or from whom did you **mostly** get this information? (please put a cross ✗ for **<u>three</u>** of the options below)	
School teacher	
Mother	
Father	
Brother	
Sister	
Other family members	
Friends	
Health worker (nurse or doctor)	
Books/magazines	
Films/Videos	
Internet	
Other (specify)_____________	
27. From whom or where, would you **most** prefer to receive on HIV and STIs? (please put a cross ✗ for **<u>one</u>** of the options below)	
School teacher	
Mother	
Father	
Brother	
Sister	

Other family members	
Friends	
Health worker (nurse or doctor)	
Books/magazines	
Films/Videos	
Internet	
Other (specify)____________	

- **SECTION 5**
-
- **Reproductive health education at school**

28. Have you ever received reproductive health education (or life-skills education) at school? (Please select one and mark X)	Yes ☐<br>No ☐ → **GO TO QUESTION 31**
29. If **yes**, which of the topics below were covered? (please put a cross ✗ against all that apply)	
Puberty	
Reproduction and Pregnancy	
Male Reproductive System	
Female Reproductive System	
Menstruation	
Sexual Health	
Sexually Transmitted Infections	
HIV/AIDS	
Relationships	

Contraception	

30. For each topic please indicate with a cross ✗ whether the information you received at school was useful or helpful to learn about (Please answer all questions)	**Did not receive any informatio n**	**Informatio n was not useful**	**Informatio n was somewhat useful**	**Informatio n was very useful**
Puberty				
Reproduction and Pregnancy				
Male Reproductive System				
Female Reproductive System				
Menstruation				
Sexual Health				
Sexually Transmitted Infections				
HIV/AIDS				
Relationships				
Contraception				

31. From the list below, please place a cross next to the topics you would like more information about (please put a cross ✗ for all that apply)	
Puberty	
Reproduction and Pregnancy	
Male Reproductive System	
Female Reproductive System	

Menstruation	
Sexual Health	
Sexually Transmitted Infections	
HIV/AIDS	
Relationships	
Contraception	

SECTION 6

Menstruation

32. For each statement below please indicate with a cross ✗ whether you think it is true, false, or you don't know:
- (Please answer all questions)

	True	False	Don't know
Menstruation happens to girls and boys			
Menstruation is a normal process			
Menstruation is a disease			
Normally a girl menstruates about once every month			
Menstruation is controlled by hormones			
Older women eventually stop menstruating			
Menstruation signifies that a girl is biologically able to get pregnant			
It is dangerous for a girl to do physical activity during menstruation			
It is healthy for a girl to bathe during menstruation			
There are some foods that are dangerous for a girl to eat when menstruating			
Menstrual blood contains dangerous substances that can harm people's health			
Menstrual blood comes from the uterus / womb			

Pregnant women menstruate			
Menstruation is a sign that a girl has reached puberty			
Menstruation occurs if an egg has not been fertilised by sperm			

33. Which of these menstrual hygiene materials do you know about? Please put a cross ✗ next to all the materials you know:

-

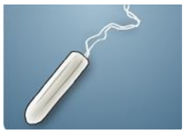 Tampon (material inserted inside the vagina to absorb blood)	
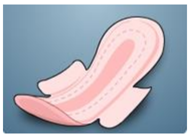 Disposable sanitary pad	
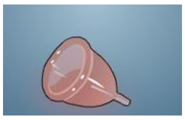 Menstrual cup (small reusable cup inserted inside the vagina to collect blood)	
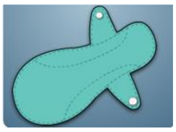 Reusable cloth that can be washed and used again	

34. People have different opinions about menstruation. For each of the opinions below please indicate using a cross ✗ whether you agree or disagree.

- (Please answer all questions)

	Agree	Disagree	Don't know
Menstruation is something that should be kept secret			
Menstruation is 'women's business' and shouldn't be discussed with men			
It is shameful or taboo to discuss menstruation			

Menstruating girls are dirty			
Menstruating girls should not be allowed to prepare or cook food			
Menstruating girls should not be allowed to enter religious buildings			
Menstruating girls should not be allowed to attend school			
Menstruation should be taught to girls at school			
Menstruation should be taught to boys at school			
A girl should be happy the first time she menstruates			
Menstruation is a sign that a girl is becoming a mature woman			

35. Have you ever received information about **menstruation?** (Please select one and mark X)	Yes ☐<br>No ☐ → **GO TO QUESTION 38**
36. How old were you when first heard about menstruation?<br><br>Age _____________ years	
37. Where or from whom did you **mostly** get information on menstruation? (please put a cross ✗ for **<u>three</u>** of the options below)	
School teacher	
Mother	
Father	
Brother	

Sister	
Other family members	
Friends	
Health worker (nurse or doctor)	
Books/magazines	
Films/Videos	
Internet	
Other (specify)_______________	
38. From whom, or where, would you **most** prefer to receive information about menstruation? (please put a cross ✗ for **one** of the options below)	
School teacher	
Mother	
Father	
Brother	
Sister	
Other family members	
Friends	
Health worker (nurse or doctor)	
Books/magazines	
Films/Videos	
Internet	

Other (specify)______________	

[Boys questionnaire will end here. Girls questionnaire will continue to the next section.]

SECTION 7

Menstrual hygiene practices and impacts

The next questions are about your experiences with menstruation and how it impacts on your life and school.

39. Have you ever had your menstrual period before (please put a cross ✗)?

Yes	139
No	140<br>141

- **If you answered <u>no</u> then you do not have to answer any of the questions below. Please hand your questionnaire back to the researcher.**

- **If you answered <u>yes</u> please answer all of the questions below:**

40. How old were you when you had your first period? Age ____________________ years

41. Had you heard about menstruation before you got your first period?

Yes	150<br>151
No	152

42. Think back to the first time you got your period. For the following statements please indicate with a cross ✗ whether you agree or disagree:
(Please answer all questions)

	Agree	Disagree	Don't know

**When I first got my period:**			
I felt happy			
I felt afraid			
I felt embarrassed			
I felt normal			
I felt proud			
I knew what was happening			
I felt prepared			
I felt ashamed			
I knew what to do to manage menstruation			
It was celebrated in my family or my community			
I felt comfortable to tell my family about it			
I felt comfortable to tell my female friends about it			
I felt comfortable to tell my male friends about it			

- **The next questions ask about your <u>most recent menstruation.</u>**

43. During your **most recent** menstruation, what material did you use to manage menstrual bleeding? Please place a cross ✗ for all the materials that you used:

New cloth	
Old cloth kept for menstruation	
Disposable sanitary pad	

Other (specify)_______________	

[Boys questionnaire will end here. Girls questionnaire will continue to the next section.]

SECTION 7

Menstrual hygiene practices and impacts

The next questions are about your experiences with menstruation and how it impacts on your life and school.

39. Have you ever had your menstrual period before (please put a cross ✗)?

Yes	139
No	140<br>141

- **If you answered no then you do not have to answer any of the questions below. Please hand your questionnaire back to the researcher.**
- **If you answered yes please answer all of the questions below:**

40. How old were you when you had your first period? Age ______________________ years

41. Had you heard about menstruation before you got your first period?

Yes	150<br>151
No	152

42. Think back to the first time you got your period. For the following statements please indicate with a cross ✗ whether you agree or disagree:
(Please answer all questions)

	Agree	Disagree	Don't know

Did not use any material (i.e. underpants only)	
Natural materials (mud, leaves)	
Other (please describe)	

45. How often did you change this material during your most recent menstruation? **Please place a cross ✗ for <u>one</u> only:**

Note that 'a day' means one day and one night (24 hours).

I didn't change the material during my period	
Every second day	
Once a day	
Twice a day	
Three times a day	
Four times a day	
Five times a day	
Other (please explain)	

46. During your most recent menstruation, how did you dispose of used materials or old materials that could no longer be reused? **Please place a cross ✗ for <u>one</u> only:**

Burn it	
Put it in the bin or landfill	
Put it in a pit latrine	
Flush it down the latrine	
Bury it	
Other (please explain)	

47. Did you reuse any materials during your most recent menstruation?

No	
Yes	

If you answer no, please skip to **question 50**

If you answer yes, please answer the questions below:

48. If you reused any materials, how did you clean the material between uses? **Please place a cross ✗ for one only:**

With water	
With water and soap	
I don't clean the material between uses	
Other (please explain)	

49. If you reused any materials, how did you dry the material between uses? **Please place a cross ✗ for one only:**

Outside in sunlight	
Inside in sunlight	
Inside not in sunlight	
Other (please explain)	

50. During your most recent menstruation, how often did you wash your hands with water and soap? **Please tick <u>one</u> only:**

Before changing menstrual hygiene material	
After changing menstrual hygiene material	
Before and after changing menstrual hygiene material	
I didn’t wash my hands with water and soap during menstruation	
I only washed my hands when I defecated or bathed	
Other (please explain)	

51. During your most recent menstruation, how often did you wash your external genitalia? **Please place a cross ✗ for <u>one</u> only:**

Only after my period had finished	
Once per day during my period	
Twice per day during my period	
Three or more times a day during my period	

I didn't wash my genitalia at all	
Other (please explain)	

52. Have you ever experienced any of the following problems while you were menstruating? Please put a cross ✗ next to all that are true for you:

Pain that stopped you doing normal activities	
Feeling tired	
Feeling dizzy or faint	
Pain when urinating	
Itching or pain of the genitals	
Difficulty concentrating	
Very heavy bleeding (having 4 or more very soaked cloths or pads a day)	

53. Have you ever missed school because of your period?

Yes	
No	

If you answer no, please skip to question **57**

54. How often do you miss school because of your period? **Please place a cross ✗ for <u>one</u> only:**

I miss school with every period	

I miss school less than every second period	
I miss school about every second periods\	

55. During your **most recent menstruation**, how many days of school did you miss because of your period? **Please place a cross ✗ for one only:**

No days missed	
1 day	
2 days	
3 days	
4 days	
5 days	
6 days	
7 days	
More than 7 days	

56. What is the **main** reason that you miss school during your period? **Please place a cross ✗ for one only:**

**I miss school during my period because:**	
I am afraid of staining my clothes	
I am afraid that I smell bad	
I am afraid of others making fun of me	
My blood flow is too heavy	
I get too much pain	

I feel too tired	
There isn't anywhere private at school to wash or change	
There isn't anywhere at school to dispose of menstrual hygiene materials	
The school latrines are dirty	
There are no latrines for girls at school	
I don't have any cloths or pads	
Religious or cultural reasons prevent me from attending school	
Other (please explain)	

57. During your most recent menstruation, did you change your material at school?

•

No	
Yes	

If you answer no, please answer the question below

If you answer yes, **please skip to question 59:**

58. What is the **main** reason why you didn't change the material at school? **Please place a cross ✗ for one only:**

Never had my period at school	
Didn't have a spare pad or cloth	
Nowhere at school to change material	
Nowhere at school to dispose of the material	

Nowhere at school to wash	
I change my material when I go home for lunch	
Other (please explain)	

59. If you did change your material at school during your most recent menstruation, where did you mostly change it? **Please place a cross ✗ for <u>one</u> only:**

Outside/open area	
Toilet/latrine/bathroom used by girls and boys	
Toilet/latrine/bathroom used by girls only	
Classroom	
Other (please explain)	

60. The following statements are about how confident you are managing menstruation. Please indicate with a cross ✗ whether you agree or disagree with each statement (Please answer all questions)

	**Agree**	**Disagree**	**Don't know**
I feel confident talking about menstruation with my mother			
I feel confident talking about menstruation with my father			

I feel confident talking about menstruation with a female relative			
I feel confident talking about menstruation with female friends			
I feel confident talking about menstruation with a female teacher			
I feel confident talking about menstruation with a male teacher			
I feel confident talking about menstruation with a health worker			
I feel confident talking about menstruation with boys			
If I got my period at school I would feel confident to go to a female teacher for help			
If I got my period at school I would feel confident to go to a male teacher for help			
I feel confident that I could manage my menstruation if I got my period while at school			
I feel confident to ask a friend or family member for a pad or other menstrual hygiene material			
I feel confident to ask a teacher for a menstrual pad or other menstrual hygiene material			
I feel confident to purchase a menstrual pad from a shop			

ADOLESCENT REPRODUCTIVE HEALTH IN MYANMAR:

FORMATIVE RESEARCH TO SUPPORT THE DELIVERY OF LIFE-SKILLS EDUCATION AND HYGIENE IMPROVEMENTS THROUGH MONASTIC SCHOOLS IN MAGWAY

FOCUS GROUP DISCUSSION: ADOLESCENT GIRLS

Materials and supplies for focus groups

- Consent forms (one copy for participants, one copy for the team)
- Coloured markers and large sheets of blank paper
- Focus Group Discussion Guide for Facilitator
- One recording device
- Batteries for recording device
- Extra tapes for recording device
- Permanent marker for marking audio tapes with FGD name, facility, and date
- Notebook for note-taking
- Refreshments

To be completed for each FGD:

Name of facilitator:		Name of note taker:	
Date (dd/mm/yy):			
Start time:		End time:	
School:		District:	

Please record the age of each FGD participant below:

Participant number	Age (years)
1	
2	
3	
4	
5	
6	

7	
8	
9	
10	
11	
12	

Has written informed consent been obtained from all participants (please cross)?

YES	
NO	

Has written informed consent from parents / guardians been obtained for all participants?

YES	
NO	

Introduction:

Facilitator: Thank you all for attending this focus group discussion today. My name is____________________. I am working with the Burnet Institute to conduct a study of young people's health in Magway. The purpose of this study is to find out about the information young people receive about reproductive health. We are also interested to understand some of the challenges that impact on girls during menstruation, particularly in schools.

This discussion is part of the study. We are interested to find out from you what information young people receive about puberty, menstruation, reproductive health. We are also interested to hear about how girls in your community manage menstruation, the challenges they face, and your opinions about how these challenges might be addressed. We are also interested to know how we can improve water and sanitation facilities in schools and provide young people with education and information about issues like puberty and menstruation.

The discussion should take around 45 minutes to 1 hour. I will ask you some questions to start the discussion, and we will take notes to record your ideas. We will also record the discussion using a tape recorder. Once we have written down the discussion from the tape recorder, the recording will be destroyed. Please remember that everything you say will be kept secret and we will not record your name on any of the notes. I would like to encourage everyone to share their ideas as everything you have to say is very important. There are no right or wrong answers so you should feel free to express yourself fully.

To make sure everyone feels comfortable to speak freely, there are a few rules for this discussion:

1. Everything we discuss here is secret, so please do not tell other people the details of this discussion after we have left this room
2. Please respect each other by not repeating information that you hear to people outside this group
3. So that everyone can speak freely I would ask that only one person talks at a time
4. Everyone's ideas and opinions are important so let us give everyone a chance to talk. Please respect others by not making other people feel uncomfortable or judging what they say
5. We would like you to stay for the whole discussion. However, if you need to leave during the discussion, please put up your hand and ask if you can leave.

At this time does anyone have any questions?

INSTRUCTIONS: BEGIN WITH AN ICE-BREAKER OR ENERGISER

Facilitator: May I begin the discussion now?

INSTRUCTIONS: FACILITATOR TURN ON THE TAPE RECORDER AND SAY CLEARLY THE DATE, TIME, FGD NUMBER, AND THE FACILITATOR'S NAME

1 Activity 1: Body mapping

We're going to start with a fun and interactive activity

Aim and description:

To trace an outline of a 'male' school student and a 'female' school student, and use creative descriptions (words and pictures) to explore how adolescent boys and girls change during puberty, and how this might impact on their ability to participate in school.

1. Ask two participants to take off their shoes and any extra clothing if you think it will interfere with drawing the outline (i.e. bulky sweater/jacket, wet shoes, etc). Ask the two participants to lie down on each on a sheet of large paper in that posture, while you trace his body outline with a pencil or grey charcoal.

2. Ask the group to trace an outline around each of the two participants. Then tell the participants that one outline drawing is a 'girl' and the other is a 'boy'. Label each drawing as a 'girl' or a 'boy'.

-

3. Ask the group to 'dress' each body with clothes to wear to school and give each a name.

QUESTION:

I'd like the group to draw the clothes this student will wear for a day at school.

4. Ask the group to illustrate on the diagrams the changes that happen to boys and girls during puberty

•

QUESTION:

As we grow form children into adults, people's bodies start going through physical changes. What kinds of changes does a boy's body go through? List them next to relevant body parts.

What kind of changes does a girl's body go through? List them next to the relevant body parts

•

5. Ask the boys to brainstorm challenges that adolescent girls and boys encounter related to puberty.

QUESTION:

What challenges does a boy encounter once he reaches puberty?

What challenges does a girl encounter once she reaches puberty?

Are these challenges the same or different for a young person who has a disability? Why?

Where do young people find out about puberty? What information are they told?

Where do young people find out about sex? What information are they told?

If not covered, ask about menstruation: One of the changes that happens to a girls when she reaches puberty is menstruation. Can anyone explain what menstruation is? Why does it happen? Where do boys find out about menstruation? What about girls?

Thank-you for your hard work on the body mapping activity. I'd like to ask you more about the information young people receive about reproductive health. There isn't any right or wrong answer, so just say whatever comes to mind. Everyone's opinions are important.

**Question**	**Probe**
What information do young people need about reproductive health?	What topics – puberty, relationships, sex, pregnancy, HIV, STIs, contraception, condoms, menstruation, etc<br><br>Do boys and girls need the same or different information?<br><br>Why do you say that?
Where should young people get this information from?	Who – parents, siblings, family, friends, teachers, health workers<br><br>How – at school, health centres, internet, books, one-on-one discussion, group discussion, etc
From what age should young people get information about reproductive health?	Why do you say that?
Is there any information that	Why do you say that?

young people shouldn’t be given?	

I would like to ask you about the reproductive health information that young people get at school. This is sometimes called ‘life-skills education’.

**Question**	**Probe**
What information do young people get about reproductive health at school?	What topics are covered – puberty, reproduction, pregnancy, STIs, HIV, safe sex, condoms, contraception, menstruation, etc<br><br>How is this information taught?<br>• What classes – or after school<br>• By who (teacher or health worker?)<br>• How often<br>• What activities are included
How useful do you think this information is?	Does it address everything that is important for young people to know?<br><br>Is it easy to understand?<br><br>Do students feel comfortable learning this information at school? Why do you say that?
How could life-skills education at	What topics should be covered?

school be improved?	Who should teach it?<br><br>How should it be taught?<br><br>• During class or after school<br>• What activities<br>• Should boys and girls be taught together or separately

Thank you for your opinions. I would now like to talk more about menstruation. Again, there are no right or wrong answers and everyone's ideas are important.<br><br>Let's think about the student (insert name) you drew here. Remember we talked about menstruation being one of the changes that would happen to her during puberty. How does her life change when she gets her first period?

Question	Probe
How does she feel when she first gets her period?	Emotions – happy, scared, embarrassed, proud? Why does she feel like this?<br><br>Is it a surprise? Would she have spoken with someone about menstruation before it happened?<br><br>Would she be prepared when she first gets her period?<br><br>Does she act differently? How?

What does she do?	Who does she talk to and why?<br><br>What does she do to manage the menstrual bleeding:<br><br>• What materials do most girls use?<br>• Where do they get them from?<br>• Are they good to use during menstruation or would girls prefer to use other materials?<br><br>Are there any other treatments or medicines or traditional medicines that she might take? What sorts of treatments? What are they for?<br><br>Who would have told her what to do during menstruation?
Do her family or friends treat her differently now that she has started menstruating?	How does her family react – is it something that is celebrated, or something kept secret?<br><br>Are there any things that she is not allowed to do when she is menstruating?<br><br>• Cooking or housework?<br>• Physical activities?<br>• Things she can't eat?<br>• Places she is not allowed to go?

Thank you for your thoughts. I would like to hear more about some of the challenges for girls who are menstruating, particularly for girls who have their period at school.

Question	Probe
What are the challenges for girls who are menstruating?	Are there any problems related to managing menstrual bleeding (access to materials, changing materials, disposing of materials)?<br><br>Are there any problems using latrines or bathrooms?<br><br>Are there any physical problems like pain, poor concentration, feeling unwell, changes in mood?<br><br>What about girls with a disability?
What about at school - are there any challenges for girls who have their period at school?	Do girls use different materials to managing menstrual bleeding at school compared with at home? Why?<br><br>Can girls access menstrual hygiene materials at school? Is there somewhere they can change menstrual hygiene materials at school?<br><br>Are girls expected to go to school when they have their period?<br><br>Do girls miss ever school when they have their period? Why do they miss school?<br><br>Does their participation in class or school activities change during their period? Why?<br><br>Of the challenges you have described, which

	ones are the most important?<br><br>What about the challenges for girls who have a disability?
Would any of her classmates and/or teachers know that she has her period?	How would they know?<br>• Behaviours in class or with friends?<br>• Latrine use?<br>• Different clothing?
How might people's behaviour towards her change if they know she has her period?<br>•	Teasing? Avoidance? Exclusion?<br><br>From teachers, family, peers, friends?
Which people at the school would she feel comfortable talking to about menstruation?	Why those people and how are they different from others?<br><br>If there are no people she would talk to, why?

2 Activity 2: Improving support for girls at school

Activity aim and description:
This activity is aimed at understanding what an ideal latrine or bathroom at school would be like for girls and what other information and support girls need at school. Participants are provided with materials to collaboratively or independently draw their ideal school latrines. The facilitator then follows up with questions about current facilities, what is missing, and what is needed.

Now let's think about how some of these challenges you have mentioned can be addressed in school. Let's start by thinking about what sanitation facilities girls need at school. I'd like you all to draw the 'perfect girls' toilet' – think about what it should look like, where it should be located, what facilities it should it have, what would make it private, how many there should be, etc.

Preparation instructions for the facilitator:

- Provide participants with sheets of paper to draw on. Make sure that the paper is accessible to everyone.
- Provide each girl with a marker and put some additional markers in a place where everyone can reach them.

Activity steps:

1. Ask girls to draw their ideal school latrine/toilet. Ask the girls to think about: Characteristics of each toilet as well as the whole block; location; distance from classrooms, privacy. They can also write labels on the drawings.

2. Once the drawing and/or writing is complete, ask the girls to share what they have drawn and to describe all of the features.

**Question**	**Probe**
Why did you draw these features?	Why are these important?<br><br>Which features are the *most* important?
What features are important for girls who are menstruating?	Why?<br><br>Is it different for girls who have a physical disability?
Does your school's latrines have any of the features that you have drawn here?	What is good about your school's latrines?<br><br>What things do you not like about your school's latrines? Would these things stop girls using them when they were menstruating? Why?

	How could your school's latrines be improved?

3. Then ask about other things that girls need at school when they are menstruating:

**Question**	**Probe**
What other supports do girls need when they have their period at school?	Support from students?<br><br>Support from teachers?<br><br>Menstrual hygiene materials provided in school?
What information about menstruation are girls given in school?	Is there other information that you think girls need? Who should provide it?<br><br>Imagine if you had a daughter – what information would you want to tell her about menstruation?

Does anyone have anything else they would like to say about menstruation and menstrual hygiene in schools?<br><br>This is the end of the discussion. Thank you for your participation. Your involvement is really important to us, and we appreciate you sharing your

ideas.

ADOLESCENT REPRODUCTIVE HEALTH IN MYANMAR:

FORMATIVE RESEARCH TO SUPPORT THE DELIVERY OF LIFE-SKILLS EDUCATION AND HYGIENE IMPROVEMENTS THROUGH MONASTIC SCHOOLS IN MAGWAY

FOCUS GROUP DISCUSSION: ADOLESCENT BOYS

Materials and supplies for focus groups

- Consent forms (one copy for participants, one copy for the team)
- Coloured markers and large sheets of blank paper
- Focus Group Discussion Guide for Facilitator
- One recording device
- Batteries for recording device
- Extra tapes for recording device
- Permanent marker for marking audio tapes with FGD name, facility, and date
- Notebook for note-taking
- Refreshments

To be completed for each FGD:

Name of facilitator:		Name of note taker:	
Date (dd/mm/yy):			
Start time:		End time:	
School:		District:	

Please record the age of each FGD participant below:

Participant number	Age (years)
1	
2	
3	
4	
5	
6	

7	
8	
9	
10	
11	
12	

Has written informed consent been obtained from all participants (please cross)?

YES	
NO	

Has written informed consent from parents / guardians been obtained for all participants?

YES	
NO	

Introduction:

Facilitator: Thank you all for attending this focus group discussion today. My name is____________________. I am working with the Burnet Institute to conduct a study of young people's health in Magway. The purpose of this study is to find out about the information young people receive about reproductive health. We are also interested to understand some of the challenges that impact on girls during menstruation, particularly in schools.

This discussion is part of the study. We are interested to find out from you what information young people receive about puberty, menstruation, reproductive health. We are also interested to hear about how girls in your community manage menstruation, the challenges they face, and your opinions about how these challenges might be addressed. We are also interested to know how we can improve water and sanitation facilities in schools and provide young people with education and information about issues like puberty and menstruation.

The discussion should take around 45 minutes to 1 hour. I will ask you some questions to start the discussion, and we will take notes to record your ideas. We will also record the discussion using a tape recorder. Once we have written down the discussion from the tape recorder, the recording will be destroyed. Please remember that everything you say will be kept secret and we will not record your name on any of the notes. I would like to encourage everyone to share their ideas as everything you have to say is very important. There are no right or wrong answers so you should feel free to express yourself fully.

To make sure everyone feels comfortable to speak freely, there are a few rules for this discussion:

6. Everything we discuss here is secret, so please do not tell other people the details of this discussion after we have left this room
7. Please respect each other by not repeating information that you hear to people outside this group
8. So that everyone can speak freely I would ask that only one person talks at a time
9. Everyone's ideas and opinions are important so let us give everyone a chance to talk. Please respect others by not making other people feel uncomfortable or judging what they say
10. We would like you to stay for the whole discussion. However, if you need to leave during the discussion, please put up your hand and ask if you can leave.

At this time does anyone have any questions?

INSTRUCTIONS: BEGIN WITH AN ICE-BREAKER OR ENERGISER

**Facilitator:** May I begin the discussion now?

INSTRUCTIONS: FACILITATOR TURN ON THE TAPE RECORDER AND SAY CLEARLY THE DATE, TIME, FGD NUMBER, AND THE FACILITATOR'S NAME

3 Activity 1: Body mapping

We're going to start with a fun and interactive activity

Aim and description:

To trace an outline of a 'male' school student and a 'female' school student, and use creative descriptions (words and pictures) to explore how adolescent boys and girls change during puberty, and how this might impact on their ability to participate in school.

6. Ask two participants to take off their shoes and any extra clothing if you think it will interfere with drawing the outline (i.e. bulky sweater/jacket, wet shoes, etc). Ask the two participants to lie down on each on a sheet of large paper in that posture, while you trace his body outline with a pencil or grey charcoal.

7. Ask the group to trace an outline around each of the two participants. Then tell the participants that one outline drawing is a 'girl' and the other is a 'boy'. Label each drawing as a 'girl' or a 'boy'.

8. Ask the group to 'dress' each body with clothes to wear to school and give each a name.

QUESTION:<br><br>I'd like the group to draw the clothes this student will wear for a day at school.

9. Ask the group to illustrate on the diagrams the changes that happen to boys and girls during puberty

•

QUESTION:<br><br>As we grow form children into adults, people's bodies start going through physical changes. What kinds of changes does a boy's body go through? List them next to relevant body parts.<br><br>What kind of changes does a girl's body go through? List them next to the relevant body parts

•

10. Ask the boys to brainstorm challenges that adolescent girls and boys encounter related to puberty.

QUESTION:<br><br>What challenges does a boy encounter once he reaches puberty?<br><br>What challenges does a girl encounter once she reaches puberty?<br><br>Are the challenges the same or different for a young person with a disability? Why?<br><br>Where do young people find out about puberty? What information are they told?<br><br>Where do young people find out about sex? What information are they told?<br><br>*If not covered, ask about menstruation:* One of the changes that happens to a girls when she reaches puberty is menstruation. Can anyone explain what menstruation is? Why does it happen? Where do boys find out about menstruation? What about girls?

Thank-you for your hard work on the body mapping activity. I'd like to ask you more about the information young people receive about reproductive health. There isn't any

right or wrong answer, so just say whatever comes to mind. Everyone's opinions are important.

Question	Probe
What information do young people need about reproductive health?	What topics – puberty, relationships, sex, pregnancy, HIV, STIs, contraception, condoms, menstruation, etc<br><br>Do boys and girls need the same or different information?<br><br>Why do you say that?
Where should young people get this information from?	Who – parents, siblings, family, friends, teachers, health workers<br><br>How – at school, health centres, internet, books, one-on-one discussion, group discussion, etc
From what age should young people get information about reproductive health?	Why do you say that?
Is there any information that young people shouldn't be given?	Why do you say that?

I would like to ask you about the reproductive health information that young people get at school. This is sometimes called 'life-skills education'.

Question	Probe
What information do young people get about reproductive health at school?	What topics are covered – puberty, reproduction, pregnancy, STIs, HIV, safe sex, condoms, contraception, menstruation, etc<br><br>How is this information taught?<br>• What classes – or after school<br>• By who (teacher or health worker?)<br>• How often<br>• What activities are included
How useful do you think this information is?	Does it address everything that is important for young people to know?<br><br>Is it easy to understand?<br><br>Do students feel comfortable learning this information at school? Why do you say that?
How could life-skills education at school be improved?	What topics should be covered?<br><br>Who should teach it?<br><br>How should it be taught?<br>• During class or after school<br>• What activities<br>• Should boys and girls be taught together

	or separately

Thank you for your opinions. I would now like to talk more about menstruation. Again, there are no right or wrong answers and everyone's ideas are important.<br><br>Let's think about the student (insert name) you drew here. Remember we talked about menstruation being one of the changes that would happen to her during puberty. How does her life change when she gets her first period?

**Question**	**Probe**
Can you describe what comes to mind when you hear the word 'menstruation'?	What words do boys use to describe it? Do these words have a good or bad meaning?
Where do boys learn about menstruation?	From whom? What are they told?<br><br>Do parents talk about menstruation, with their sons?<br><br>Do you think it is important for boys to know about menstruation? Why do you say that? What information do you think they should be given?

	Is it acceptable for boys to talk about menstruation? Why?
Do you know when a girl is menstruating? How do you know?	How does she act? Does a girl at school act differently during her period? Why do you think she acts differently?<br><br>Do girls talk to boys about menstruation?
How are girls expected to behave once they start menstruating?	Are there any changes to their daily routine? If so, what?<br><br>Are there places she cannot go? Where?<br><br>Are there places she cannot go on school grounds? Where?<br><br>Why are there restrictions?
Are girls treated differently once they start menstruation?	Are they treated differently by teachers? The community? Boys in school? Why?
Sometimes boys tease girls when they are menstruating. Can you tell me about a time when that may have happened at your school? Please don’t use people’s names, just talk about what happened.	What did they say?<br><br>Why did they do it?<br><br>How did teachers react when this happened?

4 Activity 2: water and sanitation in schools

We are going to do another activity to look at the sanitation facilities you have in school

Aim and description:

To explore current water and sanitation facilities, access for girls and boys, challenges that might face girls who are menstruating, and what could be improved.

1. Ask the participants to draw a map of their school. Ask them to indicate where the latrines are located.

Question	Probe
Who is able to use these latrines?	Is it different for boys compared with girls?<br><br>What about students who have a physical disability?
If the latrines are broken or not available, where do students go?	
What do you think about the latrines at school?	Are they clean? Do students like using them?<br><br>Is there water and soap for handwashing?

	What could be improved?
Do you think girls who have their period would use these latrines?	Why do you say that?<br><br>Is there anything that might stop girls using the latrines when they have their period?<br><br>Are there any things that need to be improved so that girls who have their period can use the latrines?
If a girl has her period at school, what happens to her?	Who can she talk to?<br><br>How does it affect her behavior at school?<br><br>What would she do to manage the bleeding (what materials might she use, where would she get these)?
Some girls face challenges at school when they are menstruating. What kinds of challenges might they face?	Difficulty keeping clean?<br><br>Focusing in school or participating in class?<br><br>Does she go home or miss school? Why?<br><br>What do you think could be done to make it easier for her?
Thank you very much for sharing your ideas. We just have a couple of questions to go.	

- Do you think fathers should tell their daughters about menstruation? If you had a daughter in the future, would you tell her about menstruation?

- Would boys like to know more about menstruation? What sort of information would boys like to receive?

- Does anyone have anything else they would like to say?

This is the end of the discussion. Thank you for your participation. Your involvement is really important to us, and we appreciate you sharing your ideas.

ADOLESCENT REPRODUCTIVE HEALTH IN MYANMAR:

FORMATIVE RESEARCH TO SUPPORT THE DELIVERY OF LIFE-SKILLS EDUCATION AND HYGIENE IMPROVEMENTS THROUGH MONASTIC SCHOOLS IN MAGWAY

IN-DEPTH INTERVIEW: ADOLESCENT GIRLS

Name of interviewer:		Date (dd/mm/yy):	
Start time:		End time:	
Name of school:		District	
Age of participant:			
Does the participant have a disability?		YES / NO	
Is the participant enrolled as a novice?		YES / NO	
Has written informed consent been obtained?		YES / NO	
Has parental consent been obtained?		YES / NO	

Opening/ rapport building questions (and follow-up probes in italics)

1. How old are you?
2. What grade are you in?

3. How long does it take you to get to school?
4. Who do you live with?
5. Do you have friends who live nearby?

6. Can you tell me a little about your school?
    - *What is your favourite subject in school?*

7. We are interested to know what young people are taught about health issues at school. In some schools, this is called 'life-skills education'. One topic that is often important to young people is puberty, that is the changes that happen to a young person when they grow from a child into an adult. What do you learn about puberty at school?
    - *What topics covered?*
    - *Who teaches these topics?*
    - *How often are they taught?*
    - *How helpful is the teaching?*
    - *Are there any topics that you think you need more information about?*
    -

    If not learned about puberty at school:

    - *How have you learned what you know? From whom?*
    - *What do you think you would need more information about?*
    - *Do you think schools should provide this information?*

8. What do you learn about reproductive health at school?
    - *What topics covered? (prompt: reproduction, sex and relationships, menstruation, STIs, HIV, pregnancy, condoms, contraception)*
    - *Who teaches these topics?*
    - *How often are they taught?*
    - *How helpful is the teaching?*
    - *Are there any topics that you think you need more information about?*
    -
    -

    If not learned about reproductive health at school:

    - *How have you learned what you know? From whom?*
    - *What do you think you would need more information about?*
    - *Do you think schools should provide this information?*
    -

9. What information do you think young people need about puberty and reproductive health?
    - *What topics?*
    - *Who should provide this information?*
    - *What information should be taught at school? How should it be taught?*

- *Should boys and girls be taught this information together or separately?*
- *What could be done to improve reproductive health education in school?*

Now we are going to move on to our discussion of more specific questions about menstruation.

10. Can you tell me about the first time you learned about menstruation?
    - *Did you learn about menstruation before/after first period?*
    - *Who told you about it (family, friends, teachers, health worker)?*
    - *What did they tell you?*
    - *What was your reaction to learning about menstruation? Why?*
    - *What more would you like to know?*
    - *Who would you like to learn this information from? Why?*

11. Can you tell me the story of the *first time* you got your period?

Context:

- *How old were you?*
- *How long ago was that?*
- *Did you know what to do?*

Attitudes/emotions*:*

- *What was your immediate reaction or feeling?*

Knowledge:

- *Did you know what it was when you got it? How?*

Resources/management/practices:

- *What did you use (pads, cloth, tissues, etc.)?*
- *Who/where did you get materials from? If you had to buy materials how much did they cost? Were there any challenges accessing these materials?*
- *Where did you go to manage your period (latrine, separate room, outside, etc.)?*

- *Was there water, soap, privacy?*

Support:

- *Did you tell anyone?*
- *If yes, who did you talk with? Why?*
- *Did you try to hide it or keep it a secret? Why?*
- *Who did you not want to know? Why?*
- *How did your family and friends react?*
- *Where there any problems that you experienced?*
- *What else was memorable about that experience?*

12. Can you tell me about a time you *had your period at school*?

- ***If she has not had a period at school before, ask what she thinks she would do if it happened (would she be prepared, how would she feel, how would she manage bleeding, show would she ask for help, how might it impact on her schooling)***
-

Context*:*

- *Did your period start at school, or did you go to school after it had already started?*
- *Were you prepared for it?*
- *Did you bring menstrual hygiene materials with you to school?*
    - *If yes, what did you bring? Where did you keep it?*
    - *If no, why not? What did you do when you realized you were menstruating?*

- Attitudes/emotions:
    - *How did you feel?*

Resources/management/practices:

- *What did you do?*
- *What clothes did you wear?*
- *What did you do and/or use that day to manage the bleeding (pads, cloth)?*
- *Do you normally use those materials?*
- *Who/where did you get them from?*
- *Where did you go to clean up?*
- *Did you use different materials when at home and school? Why?*

Support*:*

- *Did you tell anyone at school that you had your period? Who? Why this person?*
- *If you didn't have any hygiene materials at school is there someone you could go to for help?*
- *Are there menstrual hygiene materials available at school? Do you have to pay for them?*
- *Did anyone else at school know that you had your period? How did they react?*
- *Is there anyone at school who you wouldn't want to know that you had your period? Why?*
- *Have you ever been teased for having your period at school? How did it affect you?*

Impact:

- *What impact did your period have on your behaviour at school?*
    - *Prompt about fear of leakage, smell, other concerns*
    - *Stop you participating in normal activities? Why?*
    - *Have you ever had to miss school because of your period? Why?*
    - *Do you act differently in class?*

13. When you're menstruating now, who do you talk to about it (if anyone)?
    - *Friends/family/teacher/boyfriend?*
    - *Why do you talk to this person?*
    - *Why not others?*

14. Are there any activities you do not do when you're menstruating?
    - *Are there restrictions placed on you? Mobility? Cooking? Eating? Religious?*
    - *Why are there restrictions?*
    - *What are people's attitudes about girls who are menstruating in your community?*
    - *Are there things you prefer not to do?*
    - *Do you go to school?*
    - *Do you stay in school all day?*

15. Was there a time at school that you were asked to do something that you didn't feel you could do because you were menstruating? Please explain.
    - *Participating in class?*
    - *Answer questions? Write on the board?*
    - *Play games? Socialize? Sit near someone?*

16. Do you think that people treat you differently if they know you are menstruating?
    - *Family/teachers/friends/boys? If yes, how do they treat you differently?*
    - *Do girls ever get teased or bullied at school if people know they are menstruating?*

17. Are there any positive aspects of menstruating for girls? Please explain.
    - *Rest from regular housework/school work?*
    - *Other positive aspects?*

18. Are there any other problems that you have experienced during menstruation?
    - *Pain or other physical symptoms?*

19. Are there any other treatments or medicines that you take during menstruation?
    - *What are they?*
    - *What do you take them for?*

WASH at school

20. Does your school have latrines that students can use?
    - *Are there any rules about latrine use at school?*
    - *When can students use latrines? Breaks? During class?*
    - *Are latrines ever kept locked? Accessible?*
    - *If hauling water is needed, who does this?*
    - *Who cleans the latrines?*

21. What is the usual condition of the latrines at school?
    - *Are there separate latrines for boys and girls? Are there enough latrines?*
    - *Comments on: cleanliness? Smell? Privacy? Safety?*
    - *Soap/water/materials available for personal hygiene?*
    - *Is there a place to dispose of used menstrual hygiene materials?*

22. How does this affect girls when they have their period?
    - *Are the latrine facilities suitable for girls who have their period? Why / why not?*
    - *What improvements are needed?*
    - *Can girls dispose of personal menstrual hygiene materials? How?*

    If answered no:

    - *Where do students go to urinate? Defecate?*
    - *What about girls who are menstruating – what do they do?*

Closing questions

23. If you could give advice to your little sister/cousin before she starts menstruating to help her, what would you say?

    - *Looking back, what would you have liked to have known about menstruation before you got your first*

period?
- *What information do girls need about menstruation?*
- *What information should be provided in school?*

24. What could be done at school to better support girls who are menstruating?

25. What could parents/families do to support their girls?

This is the end of the interview. Thank you very much for your valuable time and for sharing your ideas and opinions. Your contribution is very important to us and we thank you for your participation.
